# Network traits predict ecological strategies in fungi

**DOI:** 10.1038/s43705-021-00085-1

**Published:** 2022-01-05

**Authors:** C. A. Aguilar-Trigueros, L. Boddy, M. C. Rillig, M. D. Fricker

**Affiliations:** 1 Freie Universität Berlin, Institut für Biologie, Altensteinstraße 6, 14195 Berlin, Germany; 2 Berlin-Brandenburg Institute of Advanced Biodiversity Research (BBIB), 14195 Berlin, Germany; 3 Department of Biological and Environmental Science, University of Jyväskylä, P.O. Box 35, FI-40014 Jyväskylä, Finland; 4 School of Biosciences, Sir Martin Evans Building, Cardiff University, CF10 3AX Cardiff, UK; 5 Department of Plant Sciences, University of Oxford, South Parks Road, OX1 3RB Oxford, UK

**Keywords:** Microbial ecology, Fungal ecology

## Abstract

Colonization of terrestrial environments by filamentous fungi relies on their ability to form networks that can forage for and connect resource patches. Despite the importance of these networks, ecologists rarely consider network features as functional traits because their measurement and interpretation are conceptually and methodologically difficult. To address these challenges, we have developed a pipeline to translate images of fungal mycelia, from both micro- and macro-scales, to weighted network graphs that capture ecologically relevant fungal behaviour. We focus on four properties that we hypothesize determine how fungi forage for resources, specifically: connectivity; relative construction cost; transport efficiency; and robustness against attack by fungivores. Constrained ordination and Pareto front analysis of these traits revealed that foraging strategies can be distinguished predominantly along a gradient of connectivity for micro- and macro-scale mycelial networks that is reminiscent of the qualitative ‘phalanx’ and ‘guerilla’ descriptors previously proposed in the literature. At one extreme are species with many inter-connections that increase the paths for multidirectional transport and robustness to damage, but with a high construction cost; at the other extreme are species with an opposite phenotype. Thus, we propose this approach represents a significant advance in quantifying ecological strategies for fungi using network information.

## Introduction

The body of most fungal species consists of branching filamentous hyphae and tangential hyphal fusions (i.e., anastomoses) that create a network through which nutrients and information are transported [1, 2]. This hyphal network structure is advantageous for colonizing terrestrial environments allowing fungi to connect patchily distributed ephemeral resources, such as dead organic matter in soils [3–6]. In fact, despite considerable diversification within the Fungal Kingdom and the occurrence of yeast growth forms, the vegetative mycelium network structure remains one of its defining phenotypic features [4, 5, 7]. Indeed, the highly plastic network architecture is critical for allowing fungi to respond flexibly to different spatially and temporally varying environmental conditions and biotic interactions, particularly amongst wood saprotrophic, mycorrhizal, and filamentous pathogenic fungi [8].

Despite the importance of network structure in fungi, ecologists rarely use network features as functional traits, even though fields like plant ecology already include network parameters in their trait portfolio [9, 10]. It is likely that a major challenge for the mycological community has been the limited development of methods for measuring useful network parameters in an ecological context [11–14].

Improvements in image capture and analysis now make it possible to characterize fungal mycelia over a range of length scales, from individual hyphae (micrometre scale) to macroscopic networks (metre scale). Early work measured fractal dimensions of mycelia at both micro- and macro-scales, and quantified their variation under different growth conditions or biotic interactions [15, 16]. More recently, some researchers have systematically measured simple morphological traits (e.g., hyphal number and length, spore counting or branching frequency) and continuous growth parameters (e.g., hyphal extension rate and branching rates) using automated techniques to characterize trade-offs in hyphal morphology [17], life history [18], hyphal space-searching strategies [19, 20] or to parametrize mathematical models of fungal colony growth [21–24].

Similar image processing algorithms can be adapted to capture differences in mycelial network traits that, in combination with morphological measurements of hyphae, can be used to infer the ecological strategies for fungi. Specifically, we propose traits that depend on the connectivity of the mycelial network, such as the predicted efficiency for nutrient transport, the relative construction cost of the network, and the robustness to different types of grazing, which here means the amount of damage mycelia can withstand without substantially losing connectivity [25, 26]. By connectivity, we mean the number of observed connections within a mycelium which result from branching or anastomosis. These connections can be used as a roadmap on which to predict resource transport within the mycelium. To predict transport, it is further necessary to estimate hyphal widths as well as lengths [27–30]. Width measurements have used calibrated intensity measurements [25, 26, 31, 32] or granulometry techniques [30], but are not currently included in automated image processing pipelines [21, 22, 33]. Nevertheless, by combining parameters on the connectivity of the mycelia with estimates of hyphal lengths and width, biophysical models have been developed that accurately predict within-mycelium nutrient transport dynamics in experimental settings [34, 35].

Estimation of the relative cost of the network can be made by scaling observed network traits to those from a baseline network model calculated as the minimum spanning tree (MST) [36]. This baseline model has minimal connectivity that allows at least unidirectional transport from the centre of the network to the growing tips at the foraging margin, where usually most of the resources are required (see below). Species with networks that are similar to the baseline model are predicted to follow a strategy that maximizes efficient transport of resources to the foraging margin. In contrast, mycelia that deviate from the baseline model have greater connectivity, with more investment in redundant network edges, with lower unidirectional transport capacity, but greater potential for multi-directional movement. This increased connectivity may give fungi an advantage in terms of allowing rapid resource reallocation that increases foraging ability, particularly in a patchy environment.

We include robustness measurements because the susceptibility of mycelia to grazing will influence their growth and survival. Relatively sparse networks may quickly lose connectivity after damage (low robustness), with immediate loss of resource in disconnected elements; whereas networks with greater connectivity, may be better able to contain the damage, re-route, and continue growing (high robustness). To investigate robustness relevant to fungal networks, we mimicked two types of attack—random attacks, typically used in other network domains, and a set of targeted attacks that are likely to reflect fungivory more realistically [37–40].

One approach to quantify such connectivity, transport, construction cost, and robustness traits is to translate the mycelium into a *weighted graph* representation using the terminology of network science [2, 41, 42]. The graph representation defines branch points, anastomoses, and tips as *nodes*, with the hyphae or cords (hyphal aggregates observed in macroscopic fungi) that connect them as *edges* of the network (see [21, 22], while the *weights* are derived from the hyphal/cord widths and lengths (e.g., [43]). Early approaches to extract a fully-weighted graph used manual delineation of nodes and edges [26, 29, 32, 43–45], which significantly limit data throughput. However, more recently intensity-independent edge enhancement and granulometry techniques have allowed automated extraction of fully weighted networks [30].

Here we developed this pipeline further to that allow automatic translation of mycelial pictures into weighted graphs on which our proposed network metrics can be measured. To showcase the value of our approach, we characterize network metrics across a phenotypically diverse set of saprotrophic fungi growing at both micro- and macroscopic scales and use this trait characterization to discern their foraging strategies. The fungi included ascomycetous and zygomycetous fungi that only produce networks of microscopic hyphae [46], and macroscopic networks produced by basidiomycetes that consist of mm-scale hyphal aggregates (i.e., cords) growing in compressed soil mesocosms [2, 15].

We focused on foraging because it is essential for the colonization of new habitats and exploitation of resources [47]. Furthermore, prior research has identified mycelial properties that can be used to recognize distinct foraging strategies [15, 25]. For example, Boddy [15] considers that the “phalanx-guerrilla” description used for plants can be extended to describe foraging strategies in fungi. Fungi with a “phalanx” strategy are expected to have a broad explorative margin that searches for regularly dispersed resources; while fungi with a “guerrilla” strategy have dispersed, narrow, and increasingly independent search fronts.

While this conceptual classification of foraging strategies is powerful and useful, it is challenging to recognize differences between species, and quantitative metrics of the mycelia are currently limited to space filling metrics (e.g., fractal dimension), biomass, hyphal counting, or area estimation [15, 18]. With the weighted network analysis proposed, we bring quantifiable precision to the classification of foraging strategies along major axes of variation, based on the connectivity, predicted transport, construction cost, and robustness of the network, using common ordination approaches used in functional ecology [48], which is particularly relevant for species with intermediate behaviour. Moreover, we identified the extreme foraging phenotypes (i.e., archetypes) using Pareto theory for evolutionary tradeoffs. Unlike traditional constrained ordination that describes variation and assigns it to predictive factors, the Pareto theory and statistical approach predict the traits that such archetypes may exhibit based on maximal performance to a given set of tasks [49, 50]. Overall, we believe this work will serve as a foundation for fungal ecologists to explore links between fungal-network properties and functions, and the network-trait patterns we have uncovered will aid a better understanding of these organisms.

## Materials and methods

### Fungal species

We used 12 fungi belonging to zygomycetous groups (*Mortierella sp1*, *Mortierella sp2*, *Mortierella sp3*, *Mortierella sp4*, *Mucor sp*, and *Umbelopsis sp*), Ascomycota (*Fusarium sp1*, *Fusarium sp2*, and *Alternaria sp*) (see Supplementary Table S1 for isolate accession numbers) and Basidiomycota (*Resinicium bicolour*, *Phanerochaete velutina*, and *Phallus impudicus*). The zygomycetous and ascomycete fungi were selected from a pool of 30 fungal species which were all isolated from soil samples collected in a semi-natural grassland in northeastern Germany [51] and maintained in a live culture collection at the Freie Universität Berlin. We selected these species because they had similar colony margin extension rates [17, 51], but produce distinctive mycelial phenotypes (even for species within the same genus).

The basidiomycete species are all wood-decaying fungi isolated from wood or cords and are maintained in the Cardiff culture collection. The images used came from the control treatment from a time-series study originally set up to explore fungal grazing interactions [52]. Species analyzed here were selected because they span a range of network architectures and predicted foraging strategies [53].

### Microphotography and image processing

For ascomycete and zygomycetous fungi, network parameters were extracted from images of fungal mycelium growing on water agar at 20 °C after approximately 10–14 h, during which time they reached approximately 10 mm in diameter. The mycelium originated from a water agar plug, to which 10 µl of 10% potato dextrose broth was added at the time of inoculation. Time-series pictures were automatically collected from the growing mycelia with bright-field illumination by tiling overlapping 49 images (distributed along a 7 × 7 grid) using a zoom microscope (Axio Zoom V16, Zeiss, Oberkochen) equipped with a low magnification objective (PlanNeoFluar Z 2.3x, 0.57NA) and internal optical zoom to give a pixel size of 0.79 µm. Tiled images were stitched together using Zeiss Zen Blue software. For basidiomycete fungi, images were taken of mycelium growing at 18 °C from 20 × 20 × 10 mm beech blocks across compressed soil growing for 8–12 d to reach 160–200 mm diameter (see Supplementary Material for detailed description of the pictures settings). The pixel size was between 80 and 155 µm. Thus, although images were collected from fungi at different length scales, both experimental conditions reflect the exploration phase of the mycelium from an inoculum into a nutrient poor environment. Three replicates were included for each strain of both micro- and macrofungi to ensure the ordination plots were balanced.

### Graph network representation

**Fig. 1:**
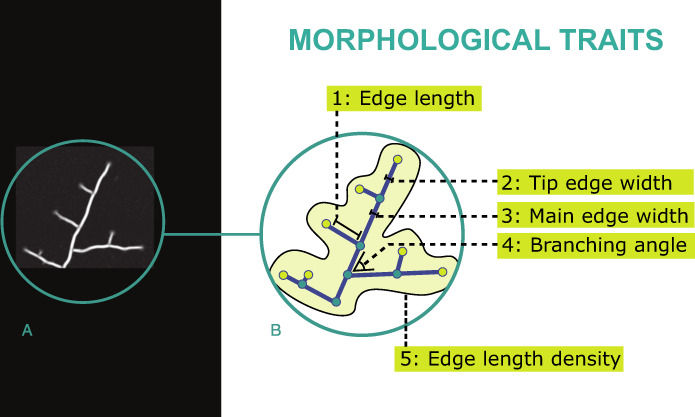
Translation of fungal mycelia into a graph object and measurement of morphological traits (traits 1–5). **A** An example of the microphotography of part of a mycelium. Using a series of image processing algorithms, this picture was translated into a network *graph* (**B**), where the *nodes* of the graph correspond to the branching points, anastomosis points (depicted in the figure as dark green circles), and hyphal tips (depicted in the figure as light green circles), while the *edges* of the graph correspond to the hyphal segments connecting the branching, anastomosis or tip points (depicted in the figure as the blue lines connecting any two nodes). Five morphological traits, measured from the real pictures using our image processing algorithms, were added to the resulting network as a vector of properties: (1) edge length, which corresponds to the hyphal length; (2) tip edge width and (3) main edge width, which corresponds to the width of the cross section of the hyphal segments connecting to a tip node or two main nodes respectively; (4) branching angle, which corresponds to the angle formed by the edges linked to a node and; (5) edge length density as the sum of all edge lengths divided by mycelial area (depicted in the figure as the enclosed grey area).

Each image was loaded into a graphical user interface (GUI, Fig. S1) and processed through a standard pipeline that included background correction, network enhancement, segmentation, skeletonization, width estimation, and network graph representation (Fig. S2) using protocols optimized across a range of biological network systems [28, 30, 54, 55]. The software package and complete manual explaining in detail the steps involved are freely available from Zenodo (10.5281/zenodo.5187932). In the resulting network graph, nodes represent hyphal tips, branching points, or anastomosis points; and edges/links represent the hyphae connecting two nodes (Fig. 1). Each edge is associated with a vector of length and width attributes, nodes with their (*x,y*) position, and branching angle. It was not possible to resolve edges within the inoculum, so these are represented as strong connections between each incident edge and a central node (termed the ‘Root’ of the network), with weights based on the Euclidean distance and the maximum edge width from the rest of the colony. This information was exported as an edge list in an.xlsx file containing all node pairs and a vector of traits, along with the position of the nodes.

The.xlsx file containing the edge list was imported into R (using the *read_excel* function from the readxl package [56] and used to construct a spatially explicit weighted network embedded in 2D space given by the Euclidean coordinates of the nodes using several functions from the igraph package for R [57] (see details in the code available in the github repo https://github.com/aguilart/Fungal_Networks). The ‘weights’ of the edges (hyphae/cords) correspond to their predicted transport resistance. For all networks, resistance scales with edge length and inversely to the edge radius to a given power. For microscopic fungi, *r*^*4*^-scaling was used (i.e., *resistance* ∝ *l*/*r*^*4*^) to simulate Poiseuille flow, whilst for macroscopic networks, *r*^*2*^-scaling was used (i.e., *resistance* ∝ *l/r*^*2*^), to reflect an increase in the number of hyphae in the cord rather than an increase in the diameter of an individual hypha [27, 44]. Thus, long and thin hyphae/cords provide higher resistance, but the precise scaling relationship differs slightly. We used the predicted transport resistance to weight hyphal segments, following a growth-induced mass flow (GIMF) model, which matches well with empirical distributions of radiolabeled nutrient movement [34, 35]. In this model, hyphae are idealized as a series of interconnected cylindrical pipes in which fluids move between two points (nodes). We recognize that this hyphal simplification is a key assumption of the model that remains to be tested for hyphal networks across scales and a diversity of species. The inoculum agar or wood block was classified as the centre of origin of the network (or “Root”) of the colony and supplied all resources needed for growth [34, 35].

**Fig. 2:**
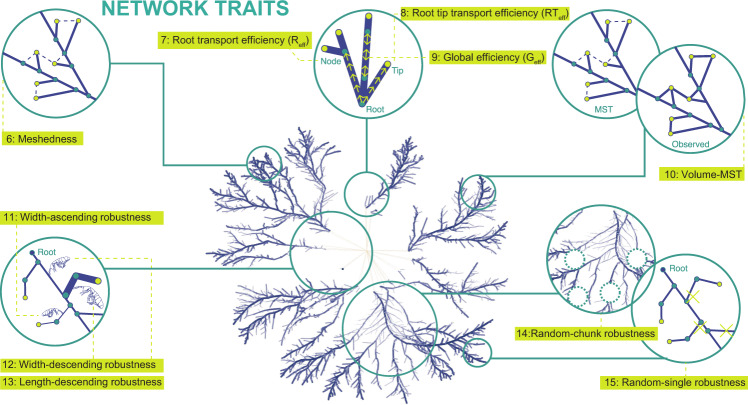
Network traits that measure distinct properties arising from the network connectivity patterns (traits 6–15). Meshedness (#6) is a topological measure based on how frequently the number of anastomosis cycles deviates from the number of such cycles in a (hypothetical) fully connected network (where all nodes are linked to their neighbours with no crossings). Root efficiency (*R*_eff_, #7) measures the expected efficiency of unidirectional transport from the inoculum (root) to any point in the mycelium; whilst Root-tip efficiency (R-T_eff_, #8) measures the expected efficiency of unidirectional transport from the inoculum (Root) to the hyphal tips (thin and long paths have low efficiency; short and wide paths have high efficiency; see Table S2 for details). Global efficiency (*G*_eff_, #9) is a measure of expected efficiency of multidirectional transport between any two points within the mycelium. Volume-MST (#10) is a measure of the relative construction cost of the network produced by comparing the observed network to a minimum spanning network (MST) that maximizes transport efficiency with the minimal number of hyphae possible. Finally, five robustness traits (#11 through #15) measure the number of edges that need to be removed to reduce the percentage of mycelium connected to the inoculum (root) to 50% (i.e., a robust mycelium is one in which a large number of edges can be removed before reducing the connected mycelium to the root to 50%). These five robustness traits refer to different patterns of hyphal removal that mimic attack by distinct types of fungivores. Note: The edges in the inoculum connecting to the root are given an arbitrary width (set to the maximum of the measured cords/hyphae) to ensure that the network is fully connected, but these values are not included in the edge statistics as they are not measured directly from the network.

To normalize the network measures, we calculated the MST of the network, using the predicted resistance to weight the edges, as a comparative approach to control for network metrics that scale with colony size (Fig. 2). Because the MST was based on hyphal resistance and not just Euclidean distances, the MST predicts the shortest path for flow from the inoculum to the tips. The MST was calculated using the function *mst* from *igraph* package for R [58].

### Traits measured

From the network graphs, we used a subset of 15 traits describing morphological and network connectivity patterns of each mycelium (which represent a minimal set of variables resulting from the filtering of over 60 variables that we originally computed, see Supplementary Information). The morphological traits (Fig. 1B) represent average measurements of hyphal attributes from the population of edges, or a single metric from the entire mycelium. These traits are mean values of: (1) hyphal length; (2) hyphal tip width; (3) main hyphae width; (4) branching angle; and (5) mycelial length density (Table S2 and Fig. 1). Network traits were derived from the connectivity patterns of the network (Fig. 2) and include: (6) meshedness, which is a measure of the topology of the network independent of edge weights; (7) Root-transport efficiency and (8) root-tip transport efficiency, which are measures of predicted unidirectional transport from the centre of origin of the network (labelled as the “Root”) to any point of the network (for 7) or just the hyphal tips (for 8); (9) global efficiency, which measures predicted multidirectional transport within the network; (10) volume-MST, which quantifies the additional construction cost of cross-linking measured against a simplified low-cost network; and five robustness traits (11 through 15), which measure the robustness of the network to different types of attack (i.e., removal of edges). Thus, in total, we measured five morphological traits and ten network traits (see Table S2 and Fig. S3 for further details).

### Statistical analysis

We performed redundancy analysis (RDA, a type-constrained multivariate ordination) to measure the extent to which variation of morphological and network traits is attributed to species identity (after standardizing all variables in units of variance to control for differences in the magnitude and measurement units among traits). We also included phylum identity as a covariate (condition) in the RDA to control for differences due to large phylogenetic distance in our set of fungi. Thus, the full RDA model was in the form *Mycelial traits* ~ *Species identity* + *Condition* (*Phylum*). To determine the extent to which the variance explained by constrained ordination deviated from a random distribution, we repeated RDA after randomizing the mycelial traits dataset (9999 times in total) and computed *F*-values and associated *p*-values (i.e., statistical significance).

Three RDA models were fitted: RDA Model 1, which included only microscopic networks (Ascomycota and zygomycetous fungi); RDA Model 2, which included only macroscopic networks (formed by cord-forming basidiomycetes). In these two models, we included both morphological and network trait types to determine the relative importance of each type of trait. Finally, we computed RDA Model 3 in which we concentrated only on network traits (after normalizing the root efficiencies (*R*_eff_ and RT_eff_) to total area). Because these network traits are independent of absolute size, we included both microscopic and macroscopic mycelia in these models. By doing so, RDA Model 3 describes a network connectivity morphospace irrespective of mycelium size and allows us to test whether there are common patterns of network structure in foraging behaviour at micro- and macro scales. This is because (a) the mycelia in both cases correspond to foraging-type networks (i.e., resources for growth are localized in the root-inoculum at the centre of the colony which supports the exploration of new resources); (b) the hydraulic transport model of transport is applicable regardless of absolute size [34, 35]; and (c) the normalized network traits included represent abstractions of how connected filaments are to each other and applies regardless of the size. Standardizations, RDA, and significance testing from the randomized approach were performed using the functions “rda” and “anova.cca” in the R package *vegan* [59].

Finally, we identified mycelial network “phenotypic archetypes” using the Pareto front approach [49, 50]. This technique identifies the vertices from the best-fitting convex hull that includes all data points in a low-dimensional ordination space. In this analysis, we used only variables normalized to total size (i.e., the same ones as in RDA Model 3) to control for variation that is driven purely by size variation. To determine how likely these archetypes are in a random distribution, we repeated this calculation after randomizing the dataset (for a total of 10,000 randomizations). Archetype identification and randomizations were performed in the open-access Pareto-front software from the Alon group (http://www.weizmann.ac.il/mcb/UriAlon/download/pareto-front-software).

## Results

### A wide diversity of fungi across different scales can be translated into a weighted graph

**Fig. 3:**
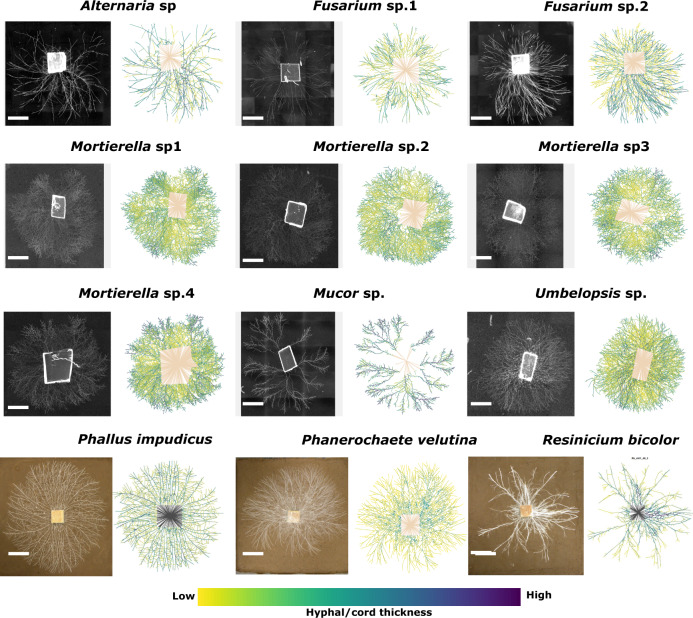
Diversity of fungal mycelia translated into weighted planar graph representations. Examples of each species displayed, with the images of the real network on the left and the corresponding graph representation on the right. In zygomycetous and ascomycete fungi (black background, first three rows), the mycelium is made up of individual hyphae growing out of water agar plugs (supplemented with potato broth at the time of inoculation) on a thin layer of water agar, while for basidiomycetes (brown background, bottom row) mycelia are made up of hyphal cords growing from wood blocks on compressed soil. The scale bar for zygomycetous and ascomycete fungi is 2 mm, and for basidiomycetes it is 40 mm. Colour scale: for microscopic fungi range goes from 1 μm (low) to 16 μm (high) hyphal width; for macroscopic fungi range goes from 0.05 mm (low) to 3 mm (high) cord width.

We translated mycelia grown on transparent agar or compressed soil into weighted graph objects for both micro- and macroscopic mycelia that span a wide taxonomic diversity (Fig. 3). In the graph representation, edges are hyphal segments that connect any two nodes whose width and length were used to predict transport resistance. The resulting fully connected weighted graphs (Fig. 3) show considerable variation across species from each of the main taxonomic clades. Visually, such variation ranges from relatively sparse networks (*Alternaria*, *Mucor*, *Resinicium*) to much denser ones (*Fusarium*, *Mortierella, Phallus,* and *Phanerochaete*) (Fig. 3). The mycelium in the inoculum cannot be resolved and is represented as a single inoculum node that is connected to all incident edges. These edges are given a weight corresponding to the maximum in the rest of the network, to ensure that the inoculum is well connected. We note that the approach adopted here cannot distinguish between true anastomoses versus crossing points—both were represented as nodes in the network.

### The distribution of morphological traits differs between species

The shape of the distribution and the summary moments of morphological traits differed among species (Fig. S4). In the case of hyphal/cord lengths, some species showed distributions close to log-normal, while others had a more-skewed distribution. Distributions of hyphal/cord width showed larger differences, in both main hyphae (i.e., internodal hyphae) and terminal hyphae (i.e., hyphal tips), with bimodal distributions observed for some species such as *Fusarium* (Fig. S4). Nevertheless, at this stage, we used the mean as a simple summary statistic for ordination.

### Network phenotypes vary along network connectivity and morphological axes

**Fig. 4:**
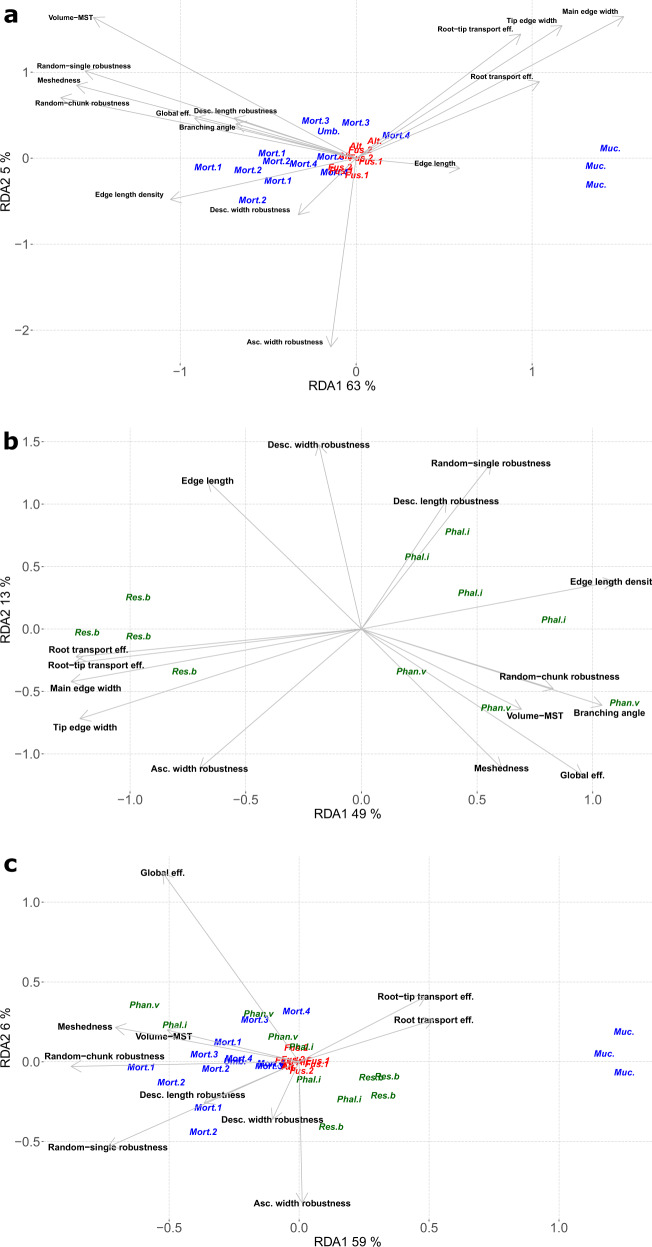
Biplots of the redundancy analysis (RDA) ordination based on mycelial traits. In all the biplots, the first two axes (RDA1 and RDA2) of each plot show the reduced space created by the morphological and network traits measured on the fungi used in this study (in parenthesis we show the amount of variation explained by each axis). The fungi are depicted with italicized abbreviations of their genera, where blue font refers to zygomycetous genera, red font ascomycetous genera, and in green font basidiomycetous genera. The position of these italicized names along the RDA axes indicates the similarity of the fungi in terms of their traits (the closer the names are located, the more similar they are respective to their traits). The name of the traits used in the analysis is abbreviated and displayed in non-italicized back font. The black arrows next to each trait name indicate the direction of increase of the trait, while the arrow length indicates the relative contribution of the trait to the first and second axis. **a** RDA of microscopic mycelia (zygomcetous and ascomycete fungi); **b** RDA of macroscopic mycelia (basidiomycete fungi); and **c** RDA including both microscopic and macroscopic mycelia.

**Fig. 5:**
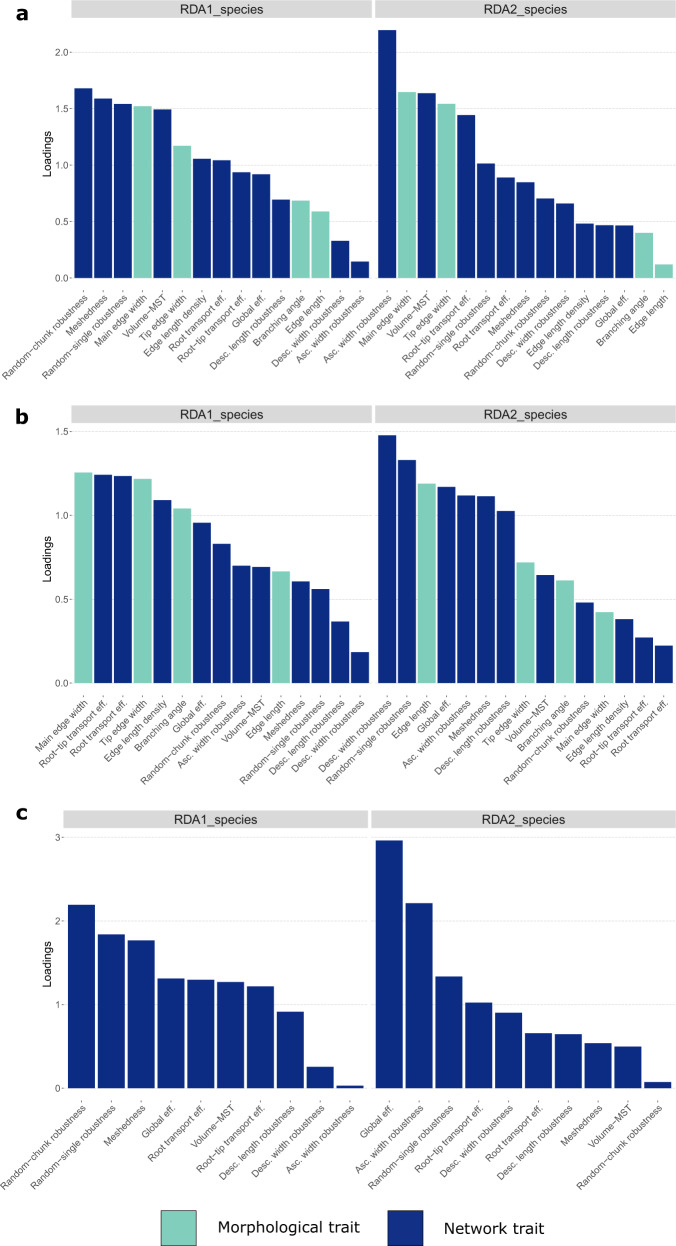
Absolute loadings (eigenvectors) of different mycelial traits for the first two main RDA axes. **a** Loadings for microscopic networks (zygomycetous fungi and ascomycetes); **b** loadings for macroscopic networks (basidiomycetes); and **c** both micro- and macro- scale networks. In all cases, loadings indicate the contribution of morphological and network traits (light and dark blue, respectively) to the first two RDA axes displayed in Fig. 4. Although each RDA axes reflect composite differences of all the traits among fungi, differences in traits with higher loading are better represented in the axes. For example, in **a** ascending width robustness has the lowest loading to RDA1, indicating that RDA1 axis does not represent well differences among species in ascending width robustness. Such differences are better represented in RDA2 where ascending width robustness has the highest loading. RDA model *c* has fewer traits than *a* and *b* because it includes only traits that are size-independent or that are size-normalized (see “Material and methods” section).

The fungal species exhibit distinct morphological and network patterns in the RDA plots (Fig. 4). Separate ordinations were used for the microscale species (Fig. 4a), the macroscale species (Fig. 4b), and a combination of both (Fig. 4c). All three RDA models explained more variation than unconstrained ordination; species-level ordination was clear even after taking into account phylum identity (when applicable), and they deviated significantly from a permutation-based random test (Tables 1 and 2). In the RDA models that included both morphological and network traits, RDA axes were largely driven by the contribution of network traits, such as topological coefficients, transport efficiencies, and robustness measures, rather than simple morphological hyphal traits, in particular for the first RDA axes (Fig. 5).

**Table 1:** Comparison of the proportion of explained variation in mycelial traits (both hyphal and network traits) between unconstrained ordination (equivalent to a PCA) and constrained ordination (RDA), in which species identity was the explanatory variable and, when applicable, Phylum identity as a conditional to control for phylogenetic relatedness.

Group/type of ordination	Correlations	Explained variation
*Zygomycetous and ascomycete fungi*		
Total	15	1
Conditional	5.13	0.34
Constrained	7.42	0.49
Unconstrained	2.45	0.16
*Basidiomycete fungi*		
Total	15.0	1.0
Constrained	9.21	0.61
Unconstrained	5.79	0.38
*All groups*		
Total	15	1
Conditional	4.30	0.37
Constrained	4.07	0.46
Unconstrained	1.63	0.16

**Table 2:** Result from permutation-based statistical test for the three RDA models based on mycelial traits (both hyphal and network traits). The first model included only microscopic mycelia (zygomycetous and ascomycete fungi), while the second model included only macroscopic mycelia (basidiomycetes). The last model included all groups of fungi. In all cases, the RDA model follows the following form: *Mycelial traits* ~ *Species identity*, with *Phylum identity* as a conditional variable (i.e., covariate) when applicable. *F*- and *p*-values were based on 9999 permutations of the constrained ordination.

Group/source of variation	Df	Variance	*F*-value	*p*-value
*Zygomycetous and ascomycete fungi*				
Species	7	7.41	6.91	0.00001
Residuals	16	1.81		
*Basidiomycete fungi*				
Species	2	9.21	6.37	6 × 10^−^^6^
Residual	8	5.78		
*All fungal groups*				
Species	9	4.07	6.65	0.00001
Residuals	24	1.6

The first RDA axis consistently explained most of the variation (40–60% depending on the model), effectively separating species along a high-to-low connectivity gradient or axis (Figs. 4 and 5). At the low-connectivity end were sparse mycelia (low mycelial density) that departed little from the MST network (low *Volume-MST* values) and were composed of consistently thick and long hyphae. These mycelia had higher Root-transport efficiencies (high values of *R*_eff_ and R-T_eff_), predicting good unidirectional transport from the centre of the colony to the tips (Fig. 4). In contrast, at the high-connectivity end were species with dense mycelia that deviated the most from the MST network (high *Volume-MST* values), indicating allocation of significant resources to cross-linking. Accordingly, they were predicted to be good at transporting resources throughout the colony regardless of directionality (high *G*_eff_*-MST*). The morphology of these fungi consisted of a high number of thin and short hyphae (low values of both hyphal lengths and widths; Fig. 4).

Robustness metrics also contributed to this first (connectivity) axis, but their importance varied between micro- and macroscale mycelia (Figs. 4 and 5), being stronger in microscopic fungi, where random-chunk robustness was the most important trait. Microscopic species at the high-connectivity end were robust against most types of attack, including random-single and random-chunk attacks as well as attacks by fungivores that prefer long or thick hyphae. The notable exception was robustness against fungivores that preferred thin hyphae—a trait that varies almost independently of the connectivity axis (Fig. 4a). In contrast, in macroscopic mycelia the contribution of robustness traits to the first axis was low, but robustness traits played a major role in the second axis. Thus, higher connectivity only correlated with higher robustness against random-chunk attacks (Fig. 4b). When considering only network traits for all fungi together, the pattern resembled that of microscopic fungi: fungi with high connectivity were more robust against most types of attack, except against fungivores that prefer thin hyphae (Fig. 4c).

The amount of explained variance in the second axis was low to moderate (6–21% for micro- and macroscopic mycelia, respectively) and it was mostly driven by robustness metrics (Figs. 4 and 5). For microscopic mycelia, this second axis was mainly driven by a single relationship, in which, microfungi with thicker hyphae were more robust to grazing by fungivores that prefer thinner hyphae. In contrast, for macroscopic mycelia, the second axis separates fungi along a trait trade-off between robustness to attack on thin cords versus robustness to random attack or attack on big cords (in terms of both length and width) (Fig. 5b). In RDA model 3, which only includes network traits, the second axis also explained little variation (6%). Here, robustness to fungivores that prefer thin hyphae also played a major role along with global transport efficiencies. That is, fungi with higher robustness against this type of attack tended to have lower global transport efficiencies.

### Limits of mycelial morphospace represented as three predicted archetypes

**Fig. 6:**
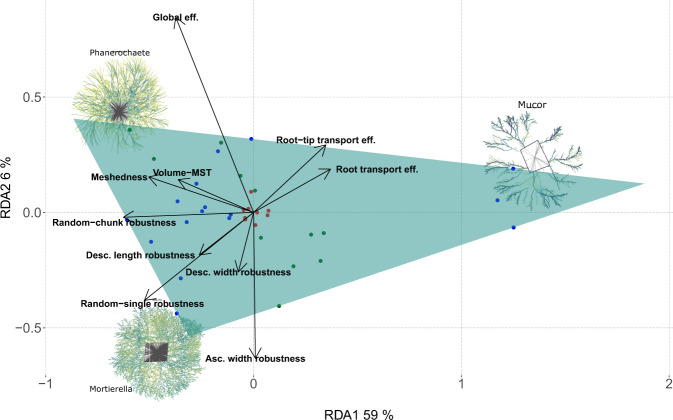
Archetype identification as vertices of the best-fitting convex hull. Each point represents a fungal colony in ordination space where blue points refer to zygomycetous fungi, red points ascomycetous fungi, and in green points basidiomycetous fungi. At each vertex, the colony morphology that is closest to the corresponding archetype is depicted. Similar to RDA ordination, the largest variation in network morphospace occurs along the first axis. This axis shows a gradient between two extremes: the left extreme (i.e., the archetype closest to *Mortierella* and *Phanerochaete ventulina*) shows high global efficiency and robustness but low root efficiency, while on the right (*Mucor*), the morphology shows an opposite pattern (low global efficiency and robustness but high root efficiency). The further separation of two archetypes at the high connectivity end on the left is driven by mild differences in global efficiency, where the archetype closest to *Phanerochaete ventulina* would have higher global efficiency than the archetype closest to Mortierella. Images of fungal mycelia are not size scaled.

Finally, using the Pareto front approach, we predict three extremes of network morphologies (archetypes) based on the combination of network traits in the ordination space (Fig. 6). These three archetypes were located mostly along the connectivity axis: one archetype morphology is located at the low connectivity end of the first axis. This archetype would consist of low global efficiency but high Root efficiency and a network that is closer to the corresponding MST (in our dataset, the closest species to this archetype is *Mucor*, Fig. 6). In contrast, the other two archetypes are located at the high connectivity end of the first axis. These two archetypes would consist of networks with high global efficiency, but lower Root efficiency, where the network is composed of more hyphae/cords than predicted by the MST, and shows high robustness to fungivores preferentially attacking big hyphae/cords. The closest species to this archetype are exemplified by *Mortierella* and *Phanerochaete velutina* (Fig. 6). The separation between these two archetypes is driven by small differences in global efficiencies, where the archetype closer to *Phanerochaete* has higher global efficiency than the archetype closest to *Mortierella*.

## Discussion

Here we argue that the properties of the mycelial networks that filamentous fungi form as they grow and search for resources can be used to understand fungal foraging strategies. We focused on network properties that we hypothesize are likely to influence how fungi overcome challenges while foraging for resources, such as connectivity, transport, construction cost, and robustness to attack. To measure these properties, we propose a set of ten network traits that, in combination with five morphological traits based on hyphal/cord dimensions, can be readily measured on both micro- and macroscopic mycelia.

### Foraging strategies can be distinguished mostly along a gradient of connectivity

We found that, at both micro- and macroscales, foraging strategies can be placed along two axes of variation. The first axis was dominant, separating foraging phenotypes based on network traits related to connectivity, transport, construction cost, and robustness. In contrast, the second gradient was almost exclusively driven by a simple morphological relationship: the thicker the hyphal/cords are on average, the higher the robustness against damage by small fungivores.

Despite the high number of traits composing the first axis, we argue that it can be understood as a *connectivity* gradient. Mycelia with high-connectivity have a high number of hyphae/cords and cross-connections per unit area. As a result, there are more potential paths for multidirectional transport. However, this connectivity also results in high construction costs. Mycelia at the low end of the connectivity gradient exhibit the opposite pattern.

Differences between micro- and macro scale networks were driven by robustness metrics. In microscopic networks, mycelia with high connectivity were robust to most types of attack. This pattern indicates that microscopic mycelia with high connectivity would remain connected despite random damage caused by grazers that can move such as collembola or isopods [60]. In contrast, among macroscopic mycelia, higher connectivity only led to higher robustness to random-chunk attack (i.e., removal of cords in chunks), while other types of robustness correlated with cord morphology (e.g., mycelium with thick cords have high robustness to attack that targets thin cords but, as expected, low robustness to attack targeting wide cords). The contrasting relationship between network connectivity and robustness traits between macro- and microscopic mycelium may be caused by the higher number of edges (i.e., connections) in the latter. Biologically such a difference makes sense, as building the edges of microscopic networks (i.e., single hyphae with short lifespan) likely has a lower metabolic cost than building the edges of macroscopic mycelia (consisting of multi-hyphal aggregated cords with a long lifespan). Thus, a positive correlation between connectivity and all types of robustness traits can only be achieved with a very high number of edges, which were not present to the same degree in the macroscopic mycelia studied here. It remains to be determined whether there is a positive correlation between connectivity and robustness in other macroscopic mycelia that have denser networks on soil [15, 53], but whose mycelia are harder to translate to a graph (data not shown). Also, future work could compare the robustness-connectivity relationship among basidiomycete, ascomycete, and zygomycetous solely at the microscopic scale.

### Comparison with other methods to characterize foraging strategies

We consider that the trait combinations of the high connectivity and low connectivity archetypes resemble, at least conceptually, the “phalanx and guerrilla” foraging types previously proposed for cord-forming fungi [15, 37]. On the one hand, the network traits behind the high connectivity archetype (i.e., high number of connections for potential multidirectional transport, high robustness, and relative construction cost) are analogous to the phalanx-like strategy that favours exploitation of the resource patch over exploration. On the other hand, the network traits behind the low connectivity archetype (i.e., high unidirectional transport, low robustness and construction cost) resemble a guerrilla strategy that favours exploration over exploitation.

Our patterns contrast with the recent study of fungal foraging behaviour of Aleklett et al. [20], in which none of fungal species used fitted unambiguously into this “phalanx-guerilla” framework. We think this seemingly contrasting results can be reconciled when looking at the scale at which trait measurements were made in both studies. In our case, we focused on network traits of a fully formed mycelium (i.e., regardless of micro- or macro- mycelia, we let the hyphae or cords assembled into a mycelium before we took measurements). In the case of Aleklett et al. [20], fungal foraging was determined by continuous measurement of space-exploration traits of individual hyphae growing in microfluidic devices (hyphal extension rate, turning angles when facing obstacles, branching rate). The fact that phalanx and guerrilla like pattern are not derived from individual hyphae trait measurements suggest that once hyphae form a connected mycelium, emergent and distinctive foraging properties arise.

Complementing hyphal level and network-level methods provides an opportunity to better understand fungal foraging strategies. For example, it would allow to determine what network traits emerge when hyphae are challenged by obstacles and constrained space. Such space-constrained environments are particularly relevant when studying mycelium consisting of hyphae (as opposed mycelium formed by cords) because it resembles more closely the habitat where hyphal networks occur (e.g., soil or plant tissue) [19, 20]. While here we used non-space-restricted systems (plates with agar or compressed sand), our pipeline can be adapted to more complex settings provided the network can be captured with high fidelity.

### Differences among species

Species identity accounted for the largest variation in network traits (46%) compared to phyla affiliation (37%, Table 1, and Fig. 4c). The largest difference between species occurs between the hyphal networks of *Mucor* sp with both the cord networks of *Phanerochaete velutina* and the hyphal networks of *Mortierella* spp. *Mucor* spp displayed a network close to a “tree-like behaviour” (formed by constant branching pattern and no anastomoses) with very low hyphal density. Such sparse tree-like mycelia is congruent with the fast growth observed in *Mucor* species [61] and their occurrence in nutrient-rich habitats [62]. In contrast, *P. velutina* and *Mortierella* spp, displayed networks traits close to the high-connectivity archetype (Fig. 6). These results are consistent with the previous description of the growth patterns of these species. For example, *Mortierella* shows frequent anastomoses [63] and dense mycelia (at least under laboratory conditions) [17] while *P. velutina* is known to increase its fractal dimension on soil mesocosm like the ones used here [15, 53].

We think that understanding the potential ecological implication of the separation in network traits between *Mucor* and *Mortierella* species deserves further research as these genera are similar in other aspects. These genera are closely related, display rapid colony growth, and a preference to grow in nutrient-rich habitats for which they are labelled “sugar fungi” [62, 64]. This discrepancy suggests that even fungi that share common habitats might show niche differentiation that is reflected or driven by their network traits.

When compared to the differences among basidiomycete and zygomycetous species in our set, the network traits of *Fusarium* sp and *Alternaria* sp (both in the Ascomycota) are very similar (Figs. 4c and 6). Such similarity is consistent with their co-occurrence in soils [65], particularly associated with the rhizosphere, and many species in both genera are common endophytes or plant pathogens [66, 67].

### Further applications of measurement of fungal network traits

We envision the integration of network traits in the repertoire of traits needed to better understand the ecological strategies in fungi. For example, fungi with low expected robustness to damage based solely on their network traits (as we examined here), might compensate for the disadvantages of limited connectivity by a multitude of alternative mechanisms. For instance, the formation of septa or glue-like proteins in response to damage could increase the resilience of micro-fungi like *Mucor* spp, by efficiently isolating the affected area, recycling the mycelia, and re-configuration of the network [68]. Alternatively, fungi can lower the chances of being grazed by decreasing the palatability of their hyphae or cords, by means of increased melanization [69], forming a thick outer rind as in rhizomorphs of *Armillaria* species [70], or by encrusting inhibitory chemicals (e.g calcium oxalate crystals) on cords of *Resinicium bicolour* [71]. As more species are included within a common comparative trait framework (i.e., measuring our proposed set of traits along with others suggested by different authors), a better definition of ecological strategies will emerge as well as determining few key traits behind such strategies as has been achieved in plant ecology [48].

Our approach can also be used to measure different individuals along a gradient of environmental conditions to determine the level of intraspecific variability and plasticity in network traits [29]. For fungi, relevant environmental gradients include climatic and edaphic factors, resource distribution (i.e., resources patchiness), inter-species competition, and grazing pressure [38, 39, 60, 72]. By tracking network traits through time, it is possible to determine to what extent age and previous conditions determine current trait values, as has been recently reported for growth traits at the hyphal [19, 20] and mycelia scales [73].

Finally, we stress that more collaborative work is needed to improve the translation of mycelial pictures into transport networks. Almost all existing approaches constrain growth to 2D and use 2D projections during analysis [21, 22, 24, 30] that may result in odd architecture with overestimation of interconnections due to hyphae overlap in slightly different planes of focus [22], which distortions connectivity and transport estimates. Measuring true connectivity and transport will require innovative techniques during image acquisition to capture the 3D structure of the fungus [23], real time recordings of hyphal flow, and the development of efficient algorithms to process these data.

In summary, our work responds to calls for developing new quantitative traits that capture the nuances of the characteristic fungal mycelial network phenotype [8, 11, 12, 14, 74]. As such, our work sets the stage for a research programme aimed at understanding the ecological diversity and evolutionary origin of the branch of life that has most successfully implemented network growth.

## Supplementary information

Supplementary figures and tables

## Data Availability

Data and code used in this paper are available in the github repo: https://github.com/aguilart/Fungal_Networks. Further information on the GUI (e.g., User Manual) available in Zenodo: 10.5281/zenodo.5187933.
