# Supplementary material for: Network traits predict ecological strategies in fungi
Source: ISME Commun. 2022 Jan 5;2:2. doi: 10.1038/s43705-021-00085-1 (PMC9723744; doi:10.1038/s43705-021-00085-1)
Supplement: Supplementary file 1 — Supplementary figures and tables [file 43705_2021_85_MOESM1_ESM.docx]

Network traits predict ecological strategies in fungi

Aguilar-Trigueros, C.A., Boddy, L., Rillig, M.C., and Fricker, M.D.

# Supplementary material


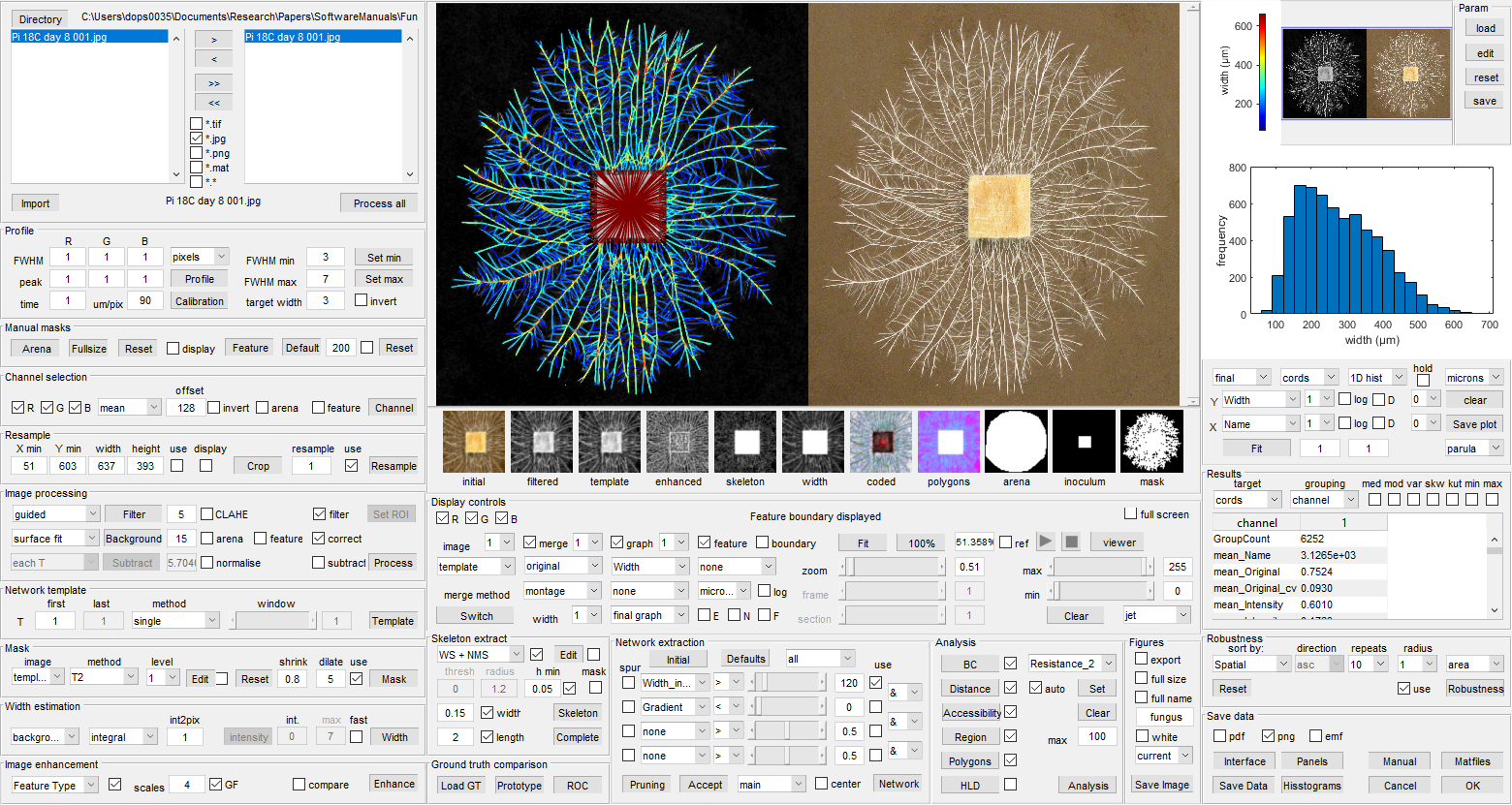


**Fig. S1 The main Fungal Network Analysis GUI.**

Images of fungal mycelia are loaded into a graphical user interface (GUI) and processed through a standard pipeline that includes noise reduction, background correction, network enhancement, segmentation, skeletonization, width estimation and network graph representation. As fungal mycelia span a range of scales, with differing contrast and noise levels depending on the imaging method, we provide a number of different curvilinear feature enhancement methods and skeletonisation algorithms to extract the network structure as a single-pixel wide binary skeleton. One of the most critical features of network analysis is to ensure that parts of the network do not become accidentally disconnected during extraction, as this may have a major impact on prediction of functional flows on the network and robustness measurements. Thus, the approach used here initially over-segments the network to ensure connectivity, calculates an initial graph and then prunes the individual edges using a combination of metrics. The pruned skeleton is then re-converted to a fully weighted graph representation, with nodes at the junctions or branch points, linked by edges with a vector of properties such as width, length and orientation. Once in a graph format, a wide range of graph theoretic measures can be calculated.

The GUI sets out the workflow for network extraction and analysis. Starting at the top-left, the processing sequence moves vertically down and then horizontally across, with key stages of the process separated out into individual panels. Typically, the button at the right-hand bottom of each panel runs the processing steps for that stage, using the parameter settings given by the controls to the left. If there are multiple steps in the stage, each one can be run individually using its own specific button. The images produced at each stage can be selected and inspected in the main display window, whilst the results for any of the measurements can be displayed in the plotting window. Results are exported in Excel format, whilst images and graphs can be exported in pdf, png or windows metafile format.

The software package and manual are available from Zenodo (DOI: <https://doi.org/10.5281/zenodo.5187932>)


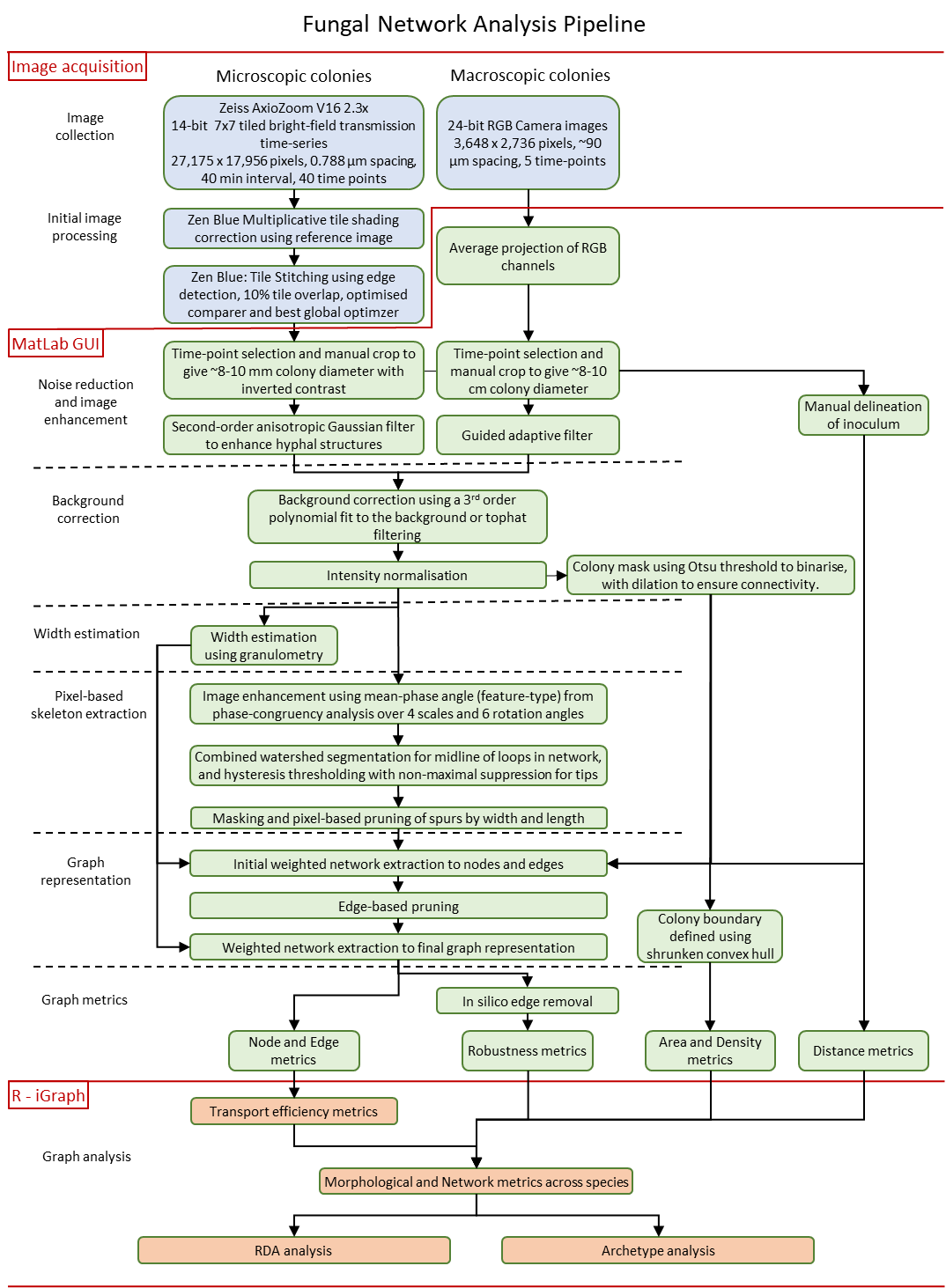


**Fig. S2 Flow diagram of the main processing steps in the network extraction and analysis pipeline.**

The initial stages of image capture differ between microscopic and macroscopic colonies (blue shading). However, subsequent processing steps within the Matlab GUI follow a similar path (green boxes). A number of network metrics are calculated within the Matlab GUI, whilst the remainder are calculated in R (orange boxes). The images and the processing parameters for each image are stored to ensure that the processing pipeline can be re-run and made available on-line at Zenodo (<https://doi.org/10.5281/zenodo.5187932>)


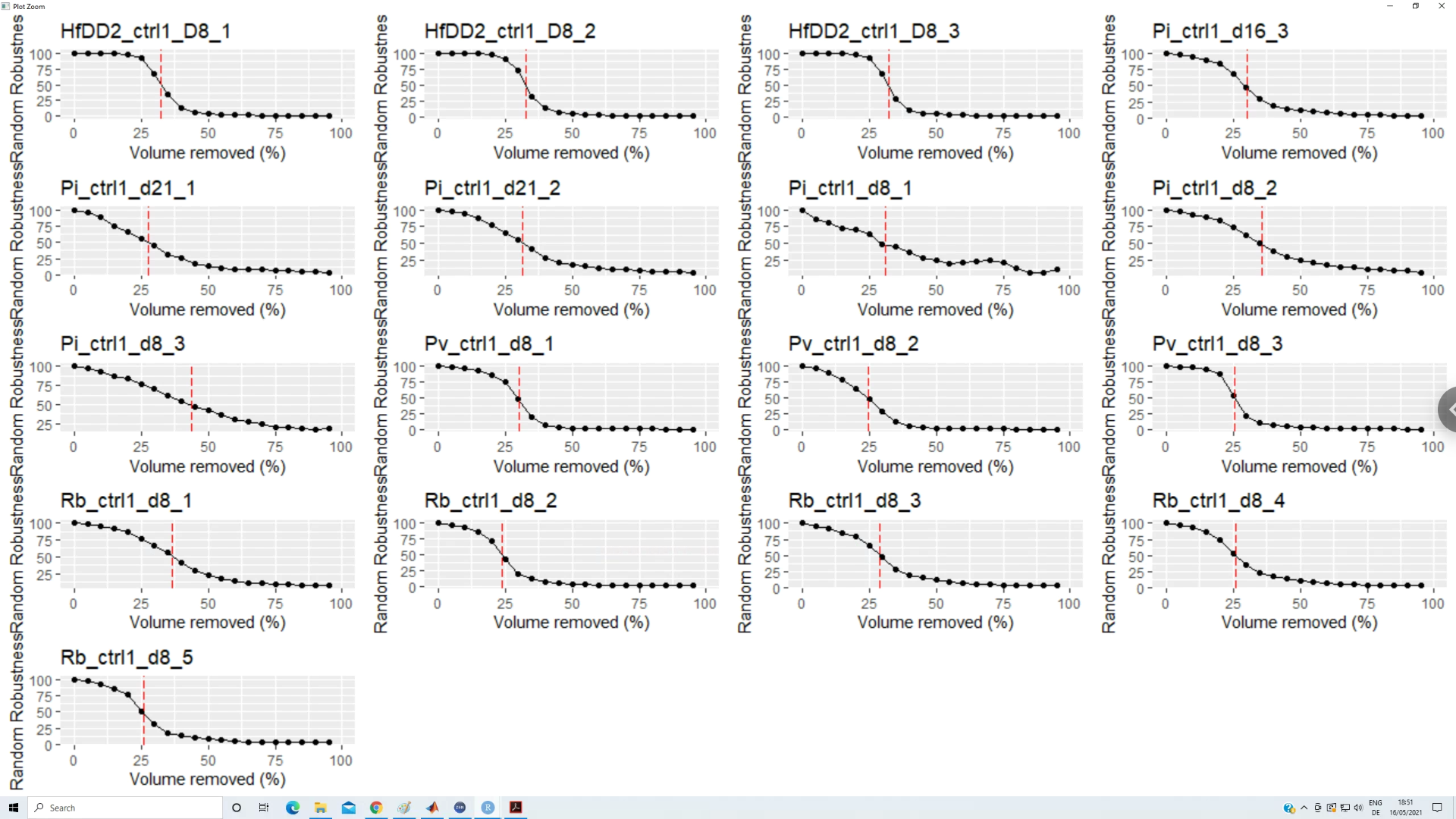


**Fig. S3 Measurement of robustness.** The x axis indicates the proportion of edges (i.e. damage) that have been removed. At each step, a sufficient number of edges are removed to sum to 5% of the original volume of the colony. Robustness (y axis) is measured as the proportion (in terms of volume) of the colony that is still connected to the inoculum after a number of edges are removed. Three criteria to select which edges to remove were used: a) in ascending or descending order (Ordered Robustness) based on some hyphal attribute (here shown hyphal width in ascending order); b) at random locations in chunks or cluster of edges (random-chunk robustness); c) at random locations of single edges (Random-single Robustness). The dotted red line marks the percentage of volume that has to be removed to reduce robustness to 50%. Here, results are shown for the Zygomycetous fungus *Mortierella sp*3.


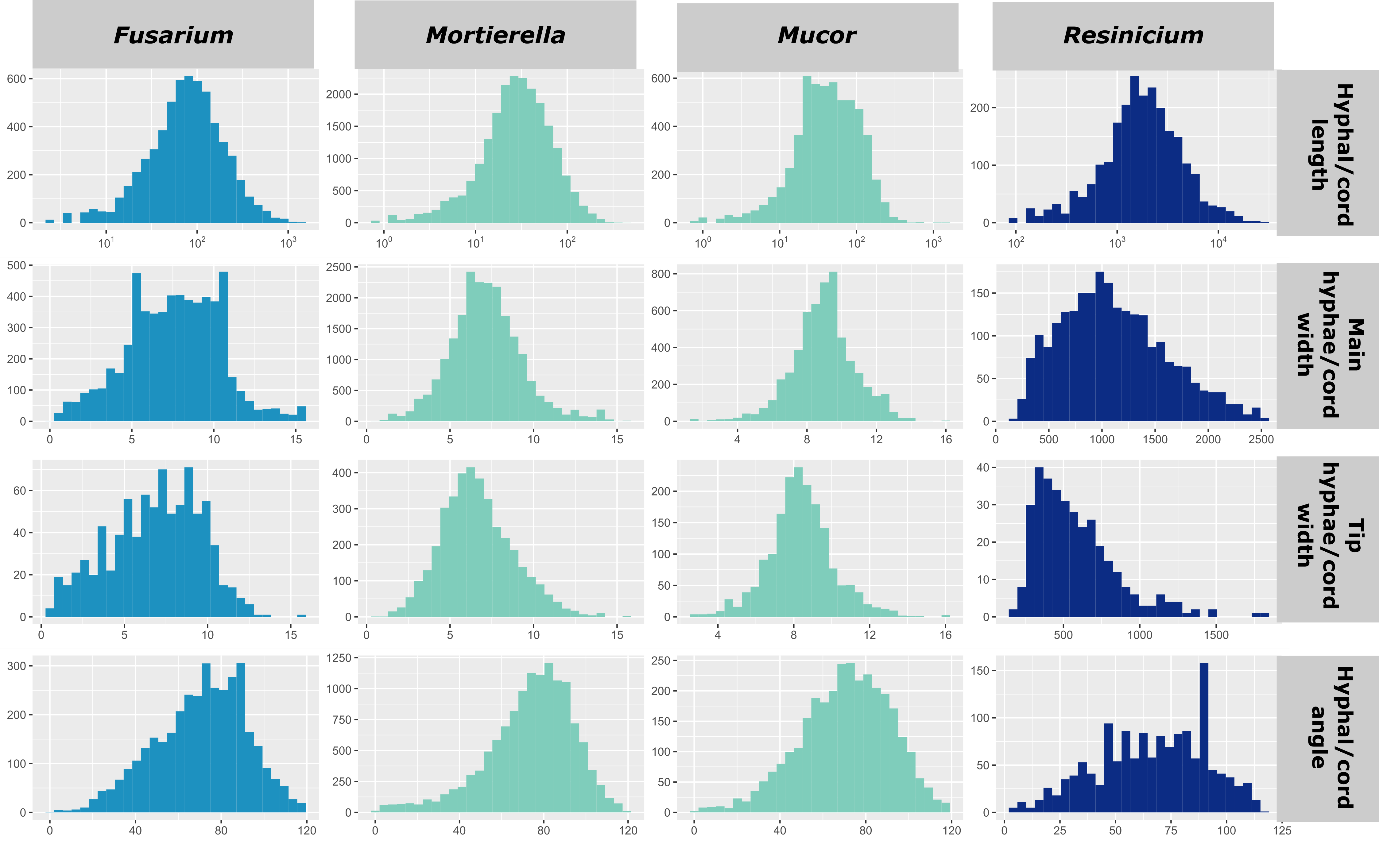


**Fig. S4** **Example typical distributions of hyphal or cord length, width (both main and tip hyphae/cord) and branching angle.** Here, the distribution from one replicate of four species: *Fusarium* (Ascomycota, in blue), *Mortierella, Mucor* (zygomycetous fungi, in light blue) and *Resinicium* (Basidiomycota, in dark blue). Lengths and width are in micrometre (length is logarithmic scale; widths are not transformed). Branching angle is given in degrees.

# Further explanation of network traits

Since most ecologists are likely unfamiliar with our network metrics (which have been adapted from other fields of network research), below we provide further explanation of our rationales for choosing them and the research supporting these traits.

- Meshnedness. This topological trait measures the ‘density’ or proportion of anastomosis cycles relative to the maximum number of cycles expected in a fully connected planar network with the same number of nodes. This maximum number of cycles can be readily calculated with simple equations based on the number on Nodes (N) and Edges (E) in the network (Table S1). This metric was originally developed to represent the topology of transport networks such as highways or railroads (Barthelemy 2018, Fricker et al 2007, Haggett and Chorley 1969), and was later coined ‘meshnedness’ in studies on ant colonies (Buhl et al 2004).
- Transport efficiencies (R_eff_, RT_eff_, G_eff_). The abstracted transport efficiency between two nodes follows the path that minimizes the hyphal resistance between them and is calculated as the inverse of the resistance-weighted shortest path i.e. a low resistance has a high predicted transport efficiency. This path resistance is measured as the sum of the resistances of the individual hyphae connecting those two points (Fig. 2). The overall network behaviour is then captured as the mycelium-wide mean of the transport efficiency between all node pairs (Global efficiency, G_eff_), from the inoculum (Root) to all other nodes (Root efficiency, R_eff_), or from the inoculum just to the hyphal tips (Root-tip efficiency, RT_eff_) (Bebber et al 2007, Latora and Marchiori 2001, Latora and Marchiori 2003) (see Table S1 for further details). We note that these measure are not normalized to the interval [0,1] using a fully connected graph as recommended by the original authors, as it is not possible to assign weights to the additional edges required to achieve full connectivity. We also note that these network metrics over-emphasize the importance of the shortest path, whilst a hydraulic model allows simultaneous parallel flows (Heaton et al 2010, Heaton et al 2012), which may be of great significance in highly cross-linked networks.
- Volume-MST. We estimated the degree to which the total volume of the observed network (as the sum of all the volumes of individual hyphae) deviated from the volume of the corresponding minimum spanning tree (MST) network as a measure of the relative cost of additional cross-links in the network. This type of comparative approach to a well-defined model is widely used in other domains network science and there are multiple models in the literature available for this purpose (Barthelemy 2018). For example, in planar networks, the connectivity of the network can be deconstructed into a sequence of geometric models, from a fully connected network (known as Delaunay triangulation), through progressively less-well-connected network models (such as the Gabriel graph and Relative Neighborhood graph) to the minimum spanning tree (MST), which minimizes the cost of building the network (Barthelemy 2018, Bebber et al 2007, Fricker et al 2007, Fricker et al 2009). However, here we restricted our analysis to comparison with the weighted MST because no good algorithms currently exist for constructing a fully connected model network that incorporates weights, such as hyphal resistance (Bebber et al 2007, Fricker et al 2007, Fricker et al 2009). As the MST keeps the minimum number of hyphae/cords needed to maintain connectivity by eliminating redundant cross connections, the ratio of the observed volume to the MST-volume provides an estimate of how much resource is allocated to cross links. (Table S1, Fig. 2).
- Robustness traits (ascending width, descending width, descending length, random-chunk and random-single). Currently, no standardized measure of the robustness of the network to potential damage (i.e. removal of edges) exists. In our case, we measured robustness as the percentage of the mycelium volume (as the sum of all the volumes of individual hyphae/cords) that was still connected to the root (inoculum) after a fixed volume of edges were removed *in silico* (Fig. 2) (Bebber et al 2007, Boddy et al 2010, Rotheray et al 2008). Edges were removed in sequential steps of cumulative sums of 5% of the total volume of the original colony before damage, running from 5% volume removed to 95% volume removed. Because robustness measured in this way resulted in profile curves (Fig. S3), we used the value at which robustness reaches 50% as a comparative metric (Fig. S3). We computed five types of robustness by simulating different types of fungivore attack or damage to the network: (1) edges were removed based on ascending width to mimic the behaviour of fungivores that preferentially attack smaller edges first (e.g. fungivorous bacteria, protist or nematodes that are likely to prefer thin and young hyphae/cords, or fungivorous mesofauna with small mouthparts that can only access thin cords, like collembola). Similarly, (2) edges were removed based on descending width to mimic fungivores that maximise return per “bite”, as in grazer soil macrofauna with larger mouth parts, like woodlice, for cord-forming fungi (A'Bear et al 2014). (3) Edges were removed based on ordered attack in descending length to reflect the probability of encounter (i.e. longer hyphae/cords are more likely to be attacked than are shorter ones). (4) We also removed edges at random, either as a group of edges (“random-chunk robustness'') that are closely located to mimic attacks by macrofauna fungivores, such as woodlice, or accidental damage. Finally, (5) we used random removal of single edges (“random-single robustness”) as a standard graph-theoretic approach (see Table S1 for further details). Ordered attacks required only one iteration because edges are uniquely ranked according to the criterion selected. For random attacks, an average of 10 repeats were used, and the 50% threshold was calculated from the average.

**Table S1** Accession number of the microscopic isolates

| **Code** | **LabID** | **Taxonomic identification** | **Phylum** | **NCBI** | **DSMZ** |
| --- | --- | --- | --- | --- | --- |
| Alt. | DF9 | *Alternaria sp.* | Ascomycota | KT582078 | DSM 100286 |
| Fus. 2 | C41 | *Fusarium redolens* | Ascomycota | KT582097 | DSM 100403 |
| Fus. 1 | FOX | *Fusarium oxysporum* | Ascomycota | KT582095 | DSM 100409 |
| Mort. 4 | DF25 | *Mortierella elongata* | Mucoromycotina | KT582072 | DSM 100407 |
| Mort. 3 | C34 | *Mortierella elongata* | Mucoromycotina | KT582092 | DSM 100402 |
| Umb. | C35 | *Umbelopsis isabellina* | Mucoromycotina | KT582093 | DSM 100331 |
| Mort. 1 | DF19 | *Mortierella alpina* | Mucoromycotina | KT582070 | DSM 100289 |
| Muc. | DF56 | *Mucor fragilis* | Mucoromycotina | KT582076 | DSM 100293 |
| Mort.2 | M | *Mortierella alpina* | Mucoromycotina | KT582067 | DSM 100285 |

**Table S2** Description of the 17 traits measured and used in our analysis. The first seven traits correspond to direct morphological measures of hyphae and entire mycelia. Then, the following 10 network traits depend directly on connectivity patterns of hyphae throughout the mycelium.

| Trait | Description |
| --- | --- |
| ***Morphological measures of hyphal traits*** | |
| (1) Mean hyphal length | This corresponds to the mean of each hyphal length in the network (defined as the measured distance between two nodes during the image processing). Given that the distribution of these lengths was left-skewed, this mean actually corresponds to log10 transformed lengths. |
|  |  |
| (2) Mean tip hyphal width | Tip hyphae are defined as hyphae connecting a hyphal tip node (which is identified as a node degree = 1). Given that the distribution of these lengths was left-skewed, this mean actually corresponds to log10 transformed widths. |
| (3) Mean main hyphal width. | Main hyphae were defined as hyphae connecting branching or anastomosis nodes. Given that the distribution of these lengths was left-skewed, this mean actually corresponds to log10 transformed widths. |
| (4) Hyphal length density | This corresponds to the total length of the mycelia (sum of hyphal/cords length in the network) divided by mycelial area |
| (5) Hyphal branch angle | The average of the minimum angles between hyphae/cords for each node with degree 3 |
|  |  |
|  |  |
| ***Network measures of mycelial traits*** | |
| (6) Meshnedness (or alpha coefficient) | Given the total number of nodes (N) and edges (E), the alpha (α) coefficient measures the proportion of cycles observed relative to the maximum number of cycles expected in a fully connected planar graph that contains the same N (Buhl et al 2004, Haggett and Chorley 1969). A cycle corresponds to a series of nodes linked to each other, like a triangle, where node a is linked to b, b to c and c to a (Figure 2). This metric is given by:  $\alpha=(E-N+1)/(2N-5)$ |
| (7) Root efficiency (R_eff_) | The mean transport efficiency from the inoculum to each node. To calculate this mean, first the paths that result in the lowest summations of resistances connecting the Root (inoculum) to each node are identified (using the Dijkstra shortest path algorithm). Root efficiency is then calculated as the mean of the sum of the inverse of the resistance for all identified paths. |
| (80) Root-tip efficiency (R-T_eff_) | The mean transport efficiency from the inoculum to each tip is calculated as described above, but includes only paths from inoculum to tips. This metric can be normalized by R_eff_ to accommodate changes in absolute colony network size. |
| (9) Global efficiency (G_eff_) | The mean transport efficiency between any two nodes. It is similar to the Root efficiency, but includes all paths connecting two nodes (not only the ones rooted to the inoculum). This metric can be normalized by R_eff_ to accommodate changes in absolute colony network size. |
| (10) Volume-MST | The ratio of the total mycelial volume to the volume of the MST calculated for the same network using the edge resistance to define the shortest paths in the MST calculation. This metric indicates the relative amount of resource allocated to cross-links not on the shortest transport path. |
| (11) Width-ascending robustness  (12) Width-descending robustness  (13) Length descending robustness | Robustness was measured as the percentage of mycelium volume that is still connected to the root after a percent volume of hyphae are removed. To mimic different types of fungivores, edges are removed in an ordered fashion depending on hyphal widths and lengths. This includes: width ascending or width descending or descending hyphal length. The comparative metric was the percentage of hyphal volume removed to reduce the robustness of the network by 50%. |
| (14) Random-chunk robustness | Random-chunk robustness was measured as the percentage mycelial volume that was still connected to the root after a percent volume of hyphae were removed based on their spatial location. That is, cluster of edges (“chunks”) were removed throughout the mycelium where the size of each individual chunk was scaled as a proportion of the area of the mycelium. The comparative metric was the percentage of hyphal volume removed to reduce the robustness of the network by 50%. |
| (15) Random single robustness | Random robustness was measured as the percentage of mycelium volume that was still connected to the inoculum-root after a percent volume of hyphae were removed at random. The comparative metric was the percentage volume removed to reduce the robustness of the network by 50%. |

REFERENCES

A'Bear AD, Jones TH, Boddy L (2014). Size matters: What have we learnt from microcosm studies of decomposer fungus–invertebrate interactions? *Soil Biol Biochem* **78:** 274-283.

Barthelemy M (2018). *Morphogenesis of spatial networks*. Springer.

Bebber DP, Hynes J, Darrah PR, Boddy L, Fricker MD (2007). Biological solutions to transport network design. *Proceedings Biological sciences / The Royal Society* **274:** 2307-2315.

Boddy L, Wood J, Redman E, Hynes J, Fricker MD (2010). Fungal network responses to grazing. *Fungal Genet Biol* **47:** 522-530.

Buhl J, Gautrais J, Solé RV, Kuntz P, Valverde S, Deneubourg JL *et al* (2004). Efficiency and robustness in ant networks of galleries. *The European Physical Journal B - Condensed Matter and Complex Systems* **42:** 123-129.

Fricker M, Boddy L, Bebber D (2007). Network organisation of mycelial fungi. *Biology of the fungal cell*. Springer. pp 309-330.

Fricker MD, Boddy L, Nakagaki T, Bebber DP (2009). Adaptive Biological Networks. In: Gross T, Sayama H (eds). *Adaptive Networks: Theory, Models and Applications*. Springer Berlin Heidelberg: Berlin, Heidelberg. pp 51-70.

Haggett P, Chorley RJ (1969). *Network analysis in geography*. Edward Arnold: London.

Heaton LLM, López E, Maini PK, Fricker MD, Jones NS (2010). Growth-induced mass flows in fungal networks. *Proceedings of the Royal Society B: Biological Sciences* **277:** 3265-3274.

Heaton LLM, López E, Maini PK, Fricker MD, Jones NS (2012). Advection, diffusion, and delivery over a network. *Physical Review E* **86:** 021905.

Latora V, Marchiori M (2001). Efficient Behavior of Small-World Networks. *Physical Review Letters* **87:** 198701.

Latora V, Marchiori M (2003). Economic small-world behavior in weighted networks. *The European Physical Journal B - Condensed Matter and Complex Systems* **32:** 249-263.

Rotheray TD, Jones TH, Fricker MD, Boddy L (2008). Grazing alters network architecture during interspecific mycelial interactions. *Fungal Ecology* **1:** 124-132.
